# Effects of the MyBFF@school obesity intervention program with nutrition education intervention on nutrition knowledge and attitude of secondary schoolchildren: a cluster randomized controlled trial

**DOI:** 10.1186/s12889-024-21090-8

**Published:** 2025-01-09

**Authors:** Rusidah Selamat, Junidah Raib, Nur Azlina Abdul Aziz, Norlida Zulkafly, Ainan Nasrina Ismail, W Nurul Ashikin W Mohamad, Ainol Aizuddin Zulkiply, Muhammad Yazid Jalaludin, Fuziah Md. Zain, Zahari Ishak, Abqariyah Yahya, Abdul Halim Mokhtar

**Affiliations:** 1 Nutrition Division, Federal Government Administrative Centre, Ministry of Health Malaysia, Level 1, Block E3, Complex E, Putrajaya, 62590 Malaysia; 2 Department of Pediatrics, Faculty of Medicine, Universiti Malaya, Kuala Lumpur, Wilayah Persekutuan Kuala Lumpur 50603 Malaysia; 3 Pediatric Department, Putrajaya Hospital, Precinct 7, Putrajaya, 62250 Malaysia; 4 FOSSLA, UCSI University, Kuala Lumpur, 56000 Malaysia; 5 Department of Social and Preventive Medicine, Faculty of Medicine, Universiti Malaya, Kuala Lumpur, 50603 Malaysia; 6 Department of Sports Medicine, Faculty of Medicine, Universiti Malaya, Kuala Lumpur, Wilayah Persekutuan Kuala Lumpur 50603 Malaysia; 7 Faculty of Sports and Exercise Science, Universiti Malaya, Kuala Lumpur, Wilayah Persekutuan Kuala Lumpur 50603 Malaysia

**Keywords:** Nutrition education intervention, Childhood obesity, Secondary schoolchildren, School-based intervention, Cluster randomized controlled trial

## Abstract

**Background:**

The increasing global and national prevalence of childhood obesity particularly among schoolchildren has warranted a more viable school-based obesity intervention. Apart from physical activity, nutrition is important in any obesity intervention package. This study examined the effects of the MyBFF@school program with nutrition education intervention (NEI) on nutrition knowledge and attitude of overweight and obese secondary schoolchildren.

**Methods:**

This is a cluster randomized controlled trial which involved schoolchildren aged 13, 14 and 16 years old from 15 out of 415 government secondary schools in central Peninsular Malaysia which were randomly assigned into six intervention (*N* = 579 schoolchildren) and nine control (*N* = 462 schoolchildren). The intervention group was given NEI consisting of a nutrition education module carried out by trained personnel for 24 weeks on top of the existing curriculum while the control group only followed the existing school curriculum by the Ministry of Education. The primary outcomes were the nutrition knowledge and attitude score. The mixed effect model taking into consideration the cluster effect was used to assess the changes of nutrition knowledge and attitude scores from baseline until 6 months.

**Results:**

Overall, there was no significant increase in the adjusted mean difference (AMD) of nutrition knowledge score (AMD = 0.33%, Confident Interval (95 CI): -4.35% to 5.01) between the intervention and control group after 6 months of intervention after controlling for nutrition knowledge score at baseline, gender, location and ethnicity. Similarly, after controlling for the nutrition attitude score at baseline, ethnicity, location and gender as well as taking into account the cluster effects, there was no significant increase on the AMD of nutrition attitude score in the overall (AMD = 0.194, (95 CI): -1.17 to 1.56) and also among girls, location (urban vs rural) and Malays. There was also no significant reduction of AMD in the nutrition attitude score among boys and non-Malays.

**Conclusion:**

MyBFF@school with NEI resulted with no significant improvement for nutrition knowledge and attitude among older schoolchildren. Therefore, to effectively impart the nutrition knowledge and change their nutrition attitude requires an in-depth study and multi-pronged and customized approach.

**Trial registration:**

Clinical trial number: NCT04155255, November 7, 2019 (Retrospective registered). National Medical Research Register: NMRR-13–439-16563. Registered July 23, 2013. The intervention program was approved by the Medical Research and Ethics Committee (MREC), Ministry of Health Malaysia and Educational Planning and Research Division (EPRD), Ministry of Education Malaysia. It was funded by the Ministry of Health Malaysia.

## Background

Childhood obesity is a global public health concern. Among children and adolescents aged 5–19 years, global trends reported in World Health Statistics 2018 have observed a significant increase in obesity from 0.8% in 1975 to 6.8% in 2016 [1]. In 2022, more than 390 million children and adolescents aged 5–19 years were overweight including obesity with increasing prevalence from 8% in 1990 to 20% in 2022 [2]. In Malaysia, the obesity prevalence among adolescents has notably increased. Findings from the National Health and Morbidity Survey showed that the prevalence of overweight Malaysian adolescents aged 10–17 years was 14.6% in 2012, but had increased to 15.6% in 2017. The prevalence of obesity increased from 12.3% to 14.8% over the same time period [3, 4]. Overweight and obese adolescents frequently suffer from body image and self-esteem problems [5]. They are also more likely to become obese as adults and suffer from non-communicable diseases such as cardiovascular diseases, type 2 diabetes mellitus, hypertension, and dyslipidemia [6].

School-based nutrition intervention is among the most effective and efficient ways to instill positive dietary changes and to influence or change the nutrition attitude of adolescents [7]. The type of school-based nutrition intervention that can be undertaken to instill positive dietary changes and to influence or change the nutrition attitude of adolescents are interventions that involve the commitment not only from the children themselves but also continuous support from the school administrators. Various studies have shown that nutrition education executed together with other obesity intervention programs effectively prevents and controls the escalation of obesity among schoolchildren [7–9]. Since these schoolchildren spent about 30% of their times in school, the control school environment would facilitate in increasing the nutrition knowledge and instilling the positive attitude. Previous school-based interventions in Asia have shown that obesity intervention is feasible and effective in a school setting. Intervention helps in changing the health behavior of children through a combination of nutrition education and physical activity, as well as by addressing psychological aspects related to the causes of childhood obesity [10–12].

The aim of this study is to assess the effects of My Body is Fit and Fabulous at School (MyBFF@school) with nutrition education intervention (NEI) on nutrition knowledge and attitude among overweight and obese secondary schoolchildren compared to schoolchildren following existing school nutrition programs.

## Methods

**Fig. 1:**
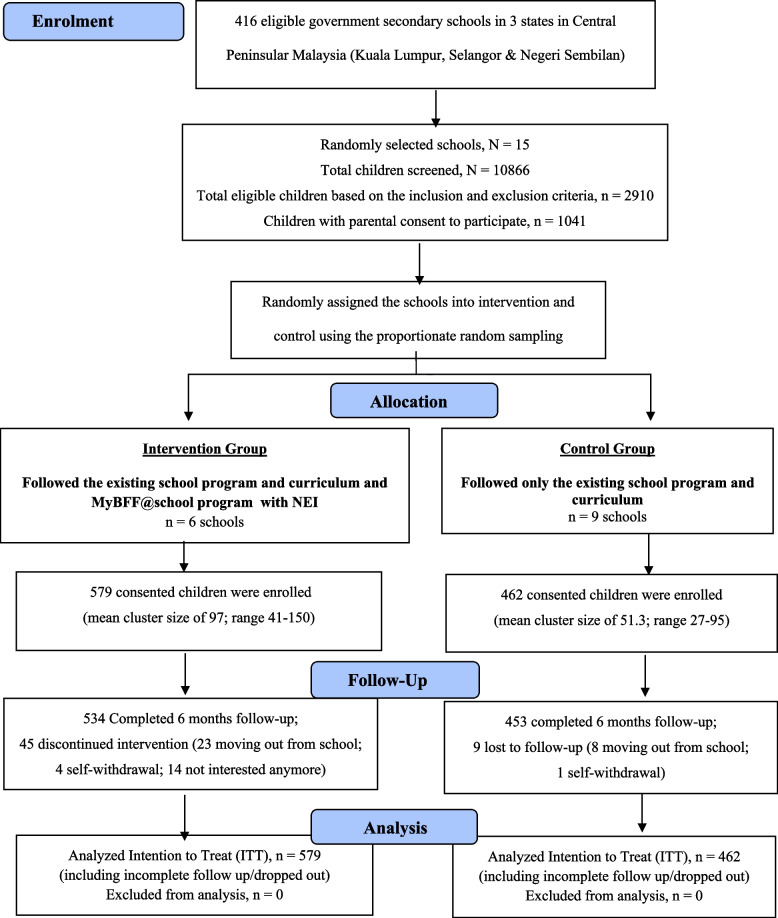
CONSORT Diagram for nutrition component in MyBFF@school

The collected data were part of the MyBFF@school obesity intervention study. This study was a school-based cluster randomized controlled trial (C-RCT) (Fig. 1). The clusters were government secondary schools with schoolchildren aged 13, 14, and 16 years in three states in central Peninsular Malaysia, namely Kuala Lumpur, Selangor and Negeri Sembilan. Schoolchildren aged 15 years old (Form 3) were excluded from this program as they were involved in major national examinations. A total of 15 out of 416 eligible government secondary schools were randomly assigned into six intervention (*n* = 579 children) and nine control schools (*n* = 462 children) taking into consideration the school type and location. The location of the schools as either in urban or rural areas was based on the current classification used by the Ministry of Education Malaysia which was adopted from the National Census by the Department of Statistics Malaysia (DOSM) 2010 [13]. Blinding of the intervention was not possible as the schools involved in the intervention or control needed to be disclosed for approval from the Ministry of Education, Malaysia apart from the staff in charge of the intervention schools needed to be trained. Recruitment of the participants within the selected clusters was based on the inclusion and exclusion criteria. The detailed methodology including the inclusion and exclusion was published by Mokhtar et al. (Mokhtar AH, Wan Mohd Zin RM, Yahya A, Md. Zain F, Selamat R, Ishak Z, Jalaludin MY: Rationale, design and methodology of My Body is Fit and Fabulous at School (MyBFF@school) Study: A multi-pronged intervention program to combat obesity among Malaysian school children). Written informed consent was obtained from parents or guardians prior to the study. Schools selected for intervention underwent the MyBFF@school with NEI, whereas control schools followed only the regular school health education syllabus. Schools involved in other obesity intervention programs were excluded from the study (Mokhtar AH, Wan Mohd Zin RM, Yahya A, Md. Zain F, Selamat R, Ishak Z, Jalaludin MY: Rationale, design and methodology of My Body is Fit and Fabulous at School (MyBFF@school) Study: A multi-pronged intervention program to combat obesity among Malaysian school children).

### Anthropometric measurements

Body impedance analyzer (InBody 720, Korea) was used to measure body weight, body fat mass, skeletal muscle mass, and percentage body fat. Body height was measured to the nearest 0.1 cm using a calibrated stadiometer (Seca 217, Germany), with the participants not wearing shoes and socks (Mokhtar AH, Wan Mohd Zin RM, Yahya A, Md. Zain F, Selamat R, Ishak Z, Jalaludin MY: Rationale, design and methodology of My Body is Fit and Fabulous at School (MyBFF@school) Study: A multi-pronged intervention program to combat obesity among Malaysian school children).

### Nutrition Education Intervention (NEI)

NEI was specifically designed to address childhood obesity and consisted of a nutrition education module (NEM). The NEM consisted of five topics, each of which was divided into several sub-topics (refer to Table 1). The topics covered included challenges in body weight loss and management, knowledge of body weight and risk factors, eating well-balanced meals, fruit and vegetable intake, increased plain water consumption, reduction in the consumption of snacks, the importance of eating breakfast, preparing a healthy meal, smart shopping, and advice for eating out. The NEI using the NEM was carried out by trained personnel who were employed under the MyBFF@school research project during co-curriculum activities after school hours for a duration of 24 weeks. Each nutrition session was conducted once every two weeks for 40–60 min per session. NEIs were adapted from the Malaysian Childhood Obesity Treatment Trial [14] and were delivered using interactive methods during practical sessions (Mokhtar AH, Wan Mohd Zin RM, Yahya A, Md. Zain F, Selamat R, Ishak Z, Jalaludin MY: Rationale, design and methodology of My Body is Fit and Fabulous at School (MyBFF@school) Study: A multi-pronged intervention program to combat obesity among Malaysian school children). These interactive NEI strategies had emphasised on hands-on competencies of nutrition education although with limited use of digital resources as shown in Table 1.

**Table 1:** Module for Nutrition Educational Intervention (NEI) for secondary schoolchildren

Topic	Sub-topic	Objective	Activity Included
**Topic 1:** Wake up Call/ Time To Act	Unit 1: Are You at Risk	To create awareness among the target groups (parents, teachers and students) on unhealthy food intake	Class teaching
	Unit 2: Challenges In Body Weight Loss and Management	To expose the target groups (parents, canteen operators, teachers and students) on challenges of weight loss management	
	Unit 3: Time To Act	To provide exposure to parents on nutrition intervention sessions that must be taken by students in MyBFF@school program	
**Topic 2:** My Body Weight/ Know My Body Weight	Unit 1: My Body Weight/ Know My Body Weight	1. To perform anthropometric measurements (height and weight) 2. To calculate Body Mass Index (BMI) and plotting the data on growth charts 3. To interpret results of BMI-for-age	Practical session on weight and height measurement, calculation and interpretation of BMI using the growth chart
**Topic 3:** Eat Well, Be Well	Unit 1: A Balancing Act/ Count To Be Fit	1. To know the concept of energy balance 2. To know the sources and total energy intake 3. To know the total energy consumption	Interactive game on getting to know the Food Pyramid and physical activity pyramid
	Unit 2: Fill In My Plate/ Healthy Eating Plan	1. To understand and apply the concept of the Malaysian Food Pyramid/Malaysian Healthy Plate 2. To understand menu planning	Practical session on how to have meal intake with the Malaysian Healthy Plate
	Unit 3: Awesome Fruits and Veggie	1. To know the importance of daily fruits and 2. vegetable consumption 3. To know the ways how to increase the intake of fruits and vegetables 4. To understand and apply the number of recommended servings	Fruit Serving Size Drawing
	Unit 4: Plain Water My Best Friend	To know the importance of drinking plain water	Interactive session to determine the amount of sugar content in beverages
	Unit 5: Less Salt and Fat/ Snack Attack	To guide the students on how to choose a healthy snack including foods with less salt and fat	Interactive session to guess the amount of salt/sodium content in food
**Topic 4:** Make a Better Life	Unit 1: Breakfast Power	1. To know the importance of breakfast 2. To know the benefit of breakfast for learning 3. To know the healthy breakfast option	Interactive session with students on the sample of healthy breakfast
**Topic 5:** MyBFF Smart Shopping, Preparing Meal Together and Eating Out	Unit 1: Smart Shopping	1. To provide guidance to the students on how to read and understand food labels as well as on how to choose food wisely 2. To provide guidance to the students on how to prepare healthy food and practise healthy eating 3. To provide guidance on how to make healthy food choices when eating out	Classroom teaching
	Unit 2: Let’s Cook/ Prepare Meal Together	To teach how to prepare healthy food	Preparing healthy balanced meals: e.g. Sandwich
	Unit 3: Eating Out: Fast Food/ Food Outlet/ School Canteen/ Hawkers	To provide information how to make healthy choice when eating out	

### Pre- and post-nutrition knowledge and attitude assessment

A similar pre-tested questionnaire was used for both pre-intervention (baseline) and post-intervention (after 6 months) assessments. Questionnaires were administered to all participants in both the intervention and control groups to assess their nutrition knowledge and attitude (KA). This nutrition knowledge questionnaire was developed for MyBFF@school by its nutrition intervention component team in the form of a 10-question true/false/don’t know nutrition knowledge questionnaire. Correct answers were given a score of 1, whereas wrong and I don’t know answers were given a score of 0. The total score for every respondent was calculated from the correct responses with a maximum of 10 points, which were then converted to a percentage. The Cronbach's alpha coefficient for the 10-item nutrition knowledge scale was 0.537. The nutrition attitude assessment consisted of 15 questions assessed on a five-point Likert scale ranging from strongly disagree (1 point) to strongly agree (5 points). An intermediate (neutral) option was allocated 3 points. The total nutrition attitude scores were also converted into percentage. The Cronbach's alpha coefficient for the 15-item nutrition attitude scale was 0.657. The total scores for the KA items were then ranked and classified into three levels: poor (< 50%), fair (between 50 and 75%), and good (more than 75%) [15].

### Statistical analysis

All data were managed using REDCap electronic data capture tools. A web-based application was designed to support data capture for research studies [16]. All analyses were performed based on the intention-to-treat for each outcome measure. Descriptive statistics were used to describe the baseline data. The chi-square test was applied to analyse the categorical data. KA assessment scores were computed as mean and standard deviation. Comparison of nutrition KA scores after 6 months and at baseline for both intervention and control groups was made using a Repeated Measure ANOVA with Greenhouse–Geisser Test. The mixed effect model taking into account the cluster effects was used to assess the changes of nutrition knowledge and attitude scores from follow up until end of 6 months. All data analyses were conducted using SPSS Statistics (Version 24; IBM Corp., Armonk, NY, USA) except for the intraclass correlation coefficient (ICC) and mixed effect model which were analysed using STATA Version 14. Results were considered statistically significant at *p* < 0.05.

## Results

### Demographic characteristics of the respondents at baseline

A total of 1041 secondary schoolchildren, with a mean age of 14.41 years, participated in this study. As shown in Table 2, the majority were Malays (79.1%), followed by Indians (14.7%), Chinese (6.0%), and others (0.3%), forming the intervention group (*n* = 579) and the control group (*n* = 462). A slight majority of the respondents were girls (58.7%) living in urban areas (64.1%). There were significant differences in the distribution of the children by location (χ^2^ = 28.39, *p* < 0.001), age groups (χ^2^ = 16.23, *p* < 0.001), and ethnicity (χ^2^ = 26.17, *p* < 0.001). There were no significant differences between the intervention and control groups in any anthropometric measures (body weight, body height, BMI-for-age z-score, skeletal muscle mass, body fat mass, and body fat percentage).

**Table 2:** Characteristics of the respondents at baseline among secondary schoolchildren

Characteristic of respondents	Intervention	Control	X^2^	*p*-value
	**(*n* = 579)**	**(*n* = 462)**		
**Gender, n (%)**				
Boys	234 (40.4)	196 (42.4)	0.43	0.53
Girls	345 (59.6)	266 (57.6)		
**Location, n (%)**^**a**^				
Urban	330 (57.0)	337 (72.9)	28.39	< 0.0001***
Rural	249 (43.0)	125 (27.1)		
**Age groups, n (%)**^**a**^				
13 years	261 (45.1)	168 (36.4)	16.23	< 0.0001***
14 years	79 (13.6)	104 (22.5)		
16 years	239 (41.3)	190 (41.1)		
**Ethnicity, n (%)**^**a**^				
Malay	486 (83.9)	337 (72.9)	26.17	< 0.0001***
Chinese	32 (5.5)	30 (6.5)		
Indian	58 (10.0)	95 (20.6)		
Others	3 (0.5)	0 (0)		
**BMI Category, n (%)**^**a**^				
Overweight	252 (43.5)	226 (48.9)	4.05	0.13
Obese	250 (43.2)	189 (40.9)		
Morbid obese	77 (13.3)	47 (10.2)		
**Anthropometric Status, mean (SD)**^**b**^				
Body weight (kg)	70.46 (15.33)	70.75 (14.83)		0.761
Body height (cm)	157.32 (8.29)	158.32 (8.29)		0.017**
BMI-for-age z-score	2.16 (0.73)	2.09 (0.71)		0.127
Skeletal muscle mass (kg)	22.98 (5.09)	23.26 (5.35)		0.377
Body fat mass (kg)	28.20 (9.45)	28.02 (9.46)		0.754
Percentage body fat	39.42 (6.90)	39.12 (7.60)		0.507

^a^Chi-square

^b^Independent T- Test

^**^p < 0.01

***p < 0.001

### Grading of nutrition KA at baseline and after 6 months of MyBFF@school with NEI

Our study indicated that the majority of schoolchildren in both the intervention and control groups had fair nutrition knowledge at baseline, albeit slightly higher in the intervention group (Table 3). At baseline, there was a significant difference in nutrition knowledge between the girls and the overall intervention group. However, after six months, there was no significant difference either in the overall group or by gender. The majority of the participants in both the intervention and control groups also had a fair nutrition attitude at baseline and after 6 months. There was also no significant difference on the grading of nutrition attitude at baseline and after 6 months between the intervention and control group. Participants from both groups, however, showed a reduction in the grading of nutrition attitude score after 6 months of NEI (Table 3).

**Table 3:** Grading nutrition KA scores in the intervention and control groups

**Parameter**	**Baseline**			**After 6 months**		
	**Intervention (*n* = 579)**	**Control (*n* = 462)**	**Chi- square test**	**Intervention (*n* = 579)**	**Control (*n* = 462)**	**Chi-square test**
**Knowledge, n (%)**						
**Overall**	*n* = 579	*n* = 462		*n* = 579	*n* = 462	
Poor (< 50.00)	55 (9.5)	58 (12.6)	*p* = 0.013	65 (11.2)	50 (10.8)	*p* = 0.433
Fair (50.00–75.00)	358 (61.8)	244 (52.8)		339 (58.5)	288 (62.3)	
Good (> 75.00)	166 (28.7)	160 (34.6)		175 (30.2)	124 (26.8)	
**Gender, n (%)**						
**Boys**	*n* = 234	*n* = 196	*p* = 0.400	*n* = 234	*n* = 196	*p* = 0.254
Poor (< 50.00)	29 (12.4)	24 (12.2)		37 (15.8)	21 (10.7)	
Fair (50.00–75.00)	140 (59.8)	106 (54.1)		142 (60.7)	131 (66.8)	
Good (> 75.00)	65 (27.8)	66 (33.7)		55 (23.5)	44 (22.4)	
**Girls**	*n* = 345	*n* = 266		*n* = 345	*n* = 266	
Poor (< 50.00)	26 (7.5)	34 (12.8)	*p* = 0.010	28 (8.1)	29 (10.9)	*p* = 0.307
Fair (50.00–75.00)	218 (63.2)	138 (51.9)		197 (57.1)	157 (59.0)	
Good (> 75.00)	101 (29.3)	94 (35.3)		120 (34.8)	80 (30.1)	
**Attitude, n (%)**						
**Overall**	*n* = 579	*n* = 462		*n* = 579	*n* = 462	
Poor (< 50.00)	39 (6.7)	32 (6.9)	*p* = 0.526	27 (4.7)	18 (3.9)	*p* = 0.820
Fair (50.00–75.00)	486 (83.9)	396 (85.7)		515 (88.9)	413 (89.4)	
Good (> 75.00)	54 (9.3)	34 (7.4)		37 (6.4)	31 (6.7)	
**Gender, n (%)**						
**Boys**	*n* = 234	*n* = 196		*n* = 234	*n* = 196	
Poor (< 50.00)	9 (3.8)	10 (5.1)	*p* = 0.374	7 (3.0)	4 (2.0)	*p* = 0.300
Fair (50.00–75.00)	197 (84.2)	170 (86.7)		214 (91.5)	174 (88.8)	
Good (> 75.00)	28 (12.0)	16 (8.2)		13 (5.6)	18 (9.2)	
**Girls**	*n* = 345	*n* = 266		*n* = 345	*n* = 266	
Poor (< 50.00)	30 (8.7)	22 (8.3)	*p* = 0.914	20 (5.8)	14 (5.3)	*p* = 0.534
Fair (50.00–75.00)	289 (83.8)	226 (85.0)		301 (87.2)	239 (89.8)	
Good (> 75.00)	26 (7.5)	18 (6.8)		24 (7.0)	13 (4.9)	

Data were presented as mean (standard deviation)

### The effects of NEI on nutrition KA scores

Table 4 shows that without controlling the nutrition knowledge score at the baseline, ethnicity, gender and location, nutrition knowledge significantly increased among girls from mean ± SD: 66.14% ± 14.13 at baseline to 68.43% ± 14.85 after 6 months in the intervention group. Among boys, nutrition knowledge scores dropped in the intervention group whereas the nutrition knowledge score slightly increased in the control group from baseline to 6 months but this change was not significant in either group. Table 4 also shows significant decreases in nutrition knowledge from baseline to 6 months for boys and rural schoolchildren in the intervention group. As noted in Table 5, although there was an increase on the adjusted mean difference (AMD) of nutrition knowledge score in the overall group, girls, urban, rural and Malays, these increases were however not significant after controlling for the nutrition knowledge score at baseline, ethnicity, location and gender while for boys and non-Malays, there was no significant reduction of AMD of nutrition knowledge score.

**Table 4:** Mean nutrition KA score in the intervention and control groups

Parameters	Intervention Group (*n* = 579)		*p* value	Control Group (*n* = 462)		*p* value
	**Baseline**	**After 6 months**		**Baseline**	**After 6 months**	
	mean (SD)	mean (SD)		mean (SD)	mean (SD)	
**Knowledge**						
Overall	65.51 (15.16)	66.21 (16.74)	0.372	65.06 (17.71)	65.26 (16.51)	0.813
Gender						
Boys	64.58 (15.88)	62.98 (18.78)	0.258	64.35 (18.21)	64.55 (16.21)	0.882
Girls	66.14 (14.63)	68.41 (14.85)	0.012*	65.57 (17.36)	65.78 (16.74)	0.854
Location						
Urban	64.80 (15.44)	66.44 (17.11)	0.127	64.65 (15.74)	65.72 (14.59)	0.265
Rural	66.46 (14.75)	65.91 (16.27)	0.633	66.14 (22.22)	64.04 (20.86)	0.273
Ethnicity						
Malay	67.48 (13.61)	67.55 (15.71)	0.926	66.82 (17.40)	67.07 (15.61)	0.795
Non-Malay	55.23 (18.42)	59.21 (20.05)	0.116	60.30 (17.75)	60.37 (17.91)	0.966
**Attitude**						
Overall	63.59 (8.95)	63.45 (7.88)	0.684	62.82 (8.93)	63.36 (7.73)	0.148
Gender						
Boys	65.08 (8.87)	63.83 (7.35)	0.020*	63.83 (8.88)	64.07 (7.70)	0.686
Girls	62.57 (8.86)	63.19 (8.21)	0.164	62.06 (8.91)	62.83 (7.73)	0.120
Location						
Urban	63.18 (9.55)	63.81 (8.37)	0.180	62.76 (8.86)	63.52 (7.55)	0.089
Rural	64.12 (8.07)	62.96 (7.15)	0.017*	62.96 (9.13)	62.93 (8.23)	0.962
Ethnicity						
Malay	63.69 (8.77)	63.38 (7.91)	0.391	62.22 (8.60)	62.76 (7.37)	0.223
Non-Malay	63.03 (9.85)	63.79 (7.71)	0.434	64.40 (9.61)	64.98 (8.46)	0.440

Data were presented as mean (standard deviation) using Repeated Measure ANOVA with Greenhouse–Geisser Test

*p < 0.05

**p < 0.01

***p < 0.001

**Table 5:** Unadjusted and adjusted nutrition knowledge and attitude score

	**DESCRIPTIVE (baseline)**				**MODEL**^**1**^			
	**Intervention**		**Control**		**Crude model**^**a**^	**Adjusted model**^**b**^		
	**N**	**Mean (SD)**	**N**	**Mean (SD)**	**Comparative** **statistic**^**c**^	**Comparative** **statistic**^**c**^	**95% CI**	**ICC**
**Nutrition knowledge score (%)**								
**Overall**	579	65.42 (15.99)	462	65.30 (18.27)	1.03	0.33	−4.36, 5.02	0.047
**Gender**								
Boys	234	63.96 (16.60)	196	64.87 (18.72)	−2.32	−2.54	−8.42, 3.34	0.038
Girls	345	66.41 (15.50)	266	65.62 (17.95)	2.81	2.12	−3.15, 7.38	0.056
**Location**								
Urban	330	64.96 (15.96)	337	64.86 (16.20)	0.18	0.22	−5.11, 5.57	< 0.000
Rural	249	66.04 (16.03)	125	66.49 (22.97)	3.16	0.21	−8.33, 8.75	< 0.000
**Ethnicity**								
Malay	486	67.28 (14.63)	337	67.08 (17.78)	1.03	1.05	−3.65, 5.75	< 0.000
Non-Malay	93	55.69 (19.07)	125	60.51 (18.77)	−1.01	−1.76	−10.39, 6.88	< 0.000
**Nutrition attitude score (%)**								
**Overall**	579	63.49 (9.11)	462	62.87 (9.01)	0.10	0.19	−1.17, 1.56	0.006
**Gender**								
Boys	234	64.93 (9.02)	196	63.75 (9.03)	−0.22	−0.01	−2.13, 2.12	0.001
Girls	345	62.52 (9.04)	266	62.22 (8.97)	0.342	0.389	−1.40, 2.18	0.008
**Location**								
Urban	330	63.09 (9.69)	337	62.80 (8.96)	0.33	0.44	−1.50, 2.39	0.010
Rural	249	64.03 (8.26)	125	63.05 (9.18)	0.11	0.19	−2.34, 2.72	1.25 × 10^–18^
**Ethnicity**								
Malay	486	63.60 (8.91)	337	62.26 (8.70)	0.55	0.55	−0.96, 2.06	0.003
Non-Malay	93	62.91 (10.08)	125	64.51 (9.66)	−1.46	−1.37	−5.08, 2.34	0.014

ICC – intra cluster (intra-school) correlation coefficient

*p < 0.05

**p < 0.01

***p < 0.001

^a^Adjusted for school mean score (nutrition knowledge and attitude) at baseline

^b^Adjusted for school mean score at baseline, gender, location, ethnicity and school group (intervention vs control)

^c^Mean difference (intervention vs control)

^d^Results from mixed effects models^c^Mean difference (intervention vs control)

The result of our study also showed that there was only a significant reduction of nutrition attitude score for boys from mean ± SD: 65.08% ± 8.8 at baseline to 63.85% ± 7.35, *p* = 0.02 after 6 months without controlling for the nutrition attitude score at the baseline, ethnicity, gender and location (Table 4). However, after controlling for the nutrition attitude score at baseline, ethnicity, gender and location as well as taking into account the cluster effects, there was no significant reduction of nutrition attitude among boys while there was no significant increase on the AMD of nutrition attitude score in the overall, girls, location (urban vs rural) and Malays (Table 5). The non-significant reduction of AMD in the nutrition attitude score was also noted among boys and non-Malays.

### Nutrition KA by item

After 6 months of participation in the MyBFF@school with NEI, correct responses for each item in the nutrition knowledge domain indicated that the highest correct scores were on the following items: 1) intake of vegetable can help in controlling body weight, 2) between six and eight glasses of water should be consumed each day, and 3) carbonated drinks (e.g., canned drinks) are not recommended because they contain a lot of sugar. On the other hand, the lowest correct scores (< 50% correct) for nutrition knowledge were for the calculation of the BMI formula and knowing that the portion size for vegetables should be at least two servings per day. In both the intervention and control groups, the highest percentage of correct scores for the nutrition knowledge was for the point that between six and eight glasses of water should be consumed each day.

For nutrition attitude, the highest mean [4.20 (1.10)] was for “I do not like drinking plain water,” with a maximum score of 5, which indicated strong agreement. The lowest mean [1.69 (0.87)] for nutrition attitude was noted for “I am worried when I am overweight,” with a minimum score of 1.

## Discussions

Our findings showed that nutrition knowledge score was only significantly increased among girls (mean ± SD: 66.14% ± 14.13) at baseline to 68.43% ± 14.85 after 6 months in the intervention group while there was no significant different for the overall, gender, ethnicity and location without controlling the nutrition knowledge score at the baseline, ethnicity, gender and location. This finding which showed girls had significantly higher nutrition knowledge scores than boys, consistent with other studies [17, 18]. In the Malaysian context and culture, this could be due to girls being more concerned about nutrition and food selection. The findings of our study are also consistent with the findings of other studies which suggested that nutrition education programs were effective in improving adolescents’ nutrition knowledge [19–22]. However, after controlling for the nutrition knowledge score at baseline, ethnicity, location and gender as well as taking into consideration the cluster effects, the findings of our study showed that among these older schoolchildren, the NEI incorporated into the MyBFF@school intervention had no significant increase on the AMD of nutrition knowledge score in the overall, girls, urban, rural and Malays. In addition, there was no significant reduction of AMD of nutrition knowledge score for boys and non-Malays.

Similarly, after controlling for the nutrition attitude score at baseline, ethnicity, location and gender taking into account the cluster effects, there was no significant difference on the increase of AMD between the intervention and control group of nutrition attitude score in the overall, girls, location (urban vs rural) and Malays. There was also no significant reduction of AMD in the nutrition attitude score among boys and non-Malays although there was a significant reduction of AMD for nutrition attitude score among boys prior to controlling for the nutrition attitude score at baseline, gender, ethnicity and location. Although our NEI was undertaken for 24 weeks with relatively short contact hours, with a total of 12 contact hours of NEI, its positive effects on the nutrition attitude of the respondents may have been limited. This was noted in our findings, whereby we observed no significant increase in the AMD in the overall nutrition attitude of the children in the intervention group as compared to the control group. This might be attributed to the lack of extensive and continuous intervention, since intervention was conducted only once every two weeks for 45–60 min per session on alternate weeks for 24 weeks. Our findings are also consistent with those of a school-based nutrition education program among junior high school students in China, which suggested that no significant change of attitude in the students was due to the fact that continuous intervention was lacking [21].

Nevertheless, other studies have shown that even short durations of nutrition intervention, if conducted often (e.g., once a week for six weeks), can have positive effects on the attitude of schoolchildren [22]. Another study by Sharif Ishak et al. among adolescents aged 13 and 14 years old showed that although there were significant improvements in the nutrition knowledge of the intervention group, improvements in the attitude were not significantly difference between the intervention and control group [23]. These two studies however did not control the nutrition knowledge and attitude score at the baseline. In this respect, it seems that the more frequent the reinforcement, the more likely that it will bring positive results in nutrition attitude. Another study by Jha et al. among school children aged between 12 and 16 years old in India also showed no statistically significant difference in the mean score of attitude for healthy diet practices in both intervention and control groups [24]. The reduction of positive nutrition attitudes could, however, result from stigmatization, since the obesity intervention in our study only involved overweight and obesity adolescents and not children of normal weight. As reported by previous study, stigmatization of obese individuals can generate health disparities and interfere with obesity intervention efforts [25].

The by-item assessment of nutrition knowledge of the adolescents showed that, despite being in the category of older adolescents attending secondary schools, a high percentage of individuals experienced difficulty in calculating their BMI. There was only a slight increase in those able to correctly answer the BMI calculation in the intervention group (from 10.5% at baseline to 13.6% after 6 months). Therefore, more practical approaches toward understanding and applying the BMI concept should be given greater emphasis, since this forms the basis of self-monitoring of body weight.

The findings of our study also showed that the majority of older school children or adolescents knew that regular intake of vegetables can help in controlling body weight, that plain water should be consumed daily, and that the intake of carbonated drinks should be reduced because of their sugar content. The findings concerning the overall attitude toward eating vegetables ranged from 3.5 to 3.7 out of a maximum of 5 points on the Likert scale. Slightly higher values in the control group indicated that they did not like to consume vegetables. A similar study conducted on middle school students in Michigan showed that students involved in a nutrition education program were more likely to report increased fruit and vegetable consumption relative to students in a control group [26]. Findings from Malaysia National and Health Morbidity Survey 2017 showed that only 9.1% of overweight adolescents and 6.0% of obese adolescents consumed an adequate amount of vegetables [5]. A similar trend was observed for those who dislike plain water, which noted a higher scale score of 4.12 to 4.20 on a 5 point Likert scale. Although the majority of the children knew the effects of consuming carbonated drink, they did not deny that they liked carbonated drinks. Various studies have shown that excessive intake of sugar from soft drinks increases energy intake, thus increasing the risk of becoming overweight or obese [27, 28]. The WHO guideline for sugar intake recommends the reduction of free sugars to less than 10% of total energy intake in both adults and children [29]. Therefore, concerted efforts have been made to reduce the intake of carbonated drinks, especially among adolescents, whereby the current Malaysian School Management Canteen Guidelines has banned the sale of carbonated drinks in school canteens.

One of the strengths of our study was this was designed as a Randomised Cluster Control Trial taking into consideration the cluster effects, location (urban vs rural) as well as the ethnicity. Therefore, the findings that could be generalized and adopted to the population. On the other hand, one of the limitations of the present study was the Cronbach’s alpha coefficients for nutrition knowledge and attitude were rather low which could possibly affect the overall consistency of the nutrition knowledge and attitude. Another limitation of the study was the MyBFF@school was lacking of direct parental involvement despite these schoolchildren had obtained the parental consent to participate in the study. Besides that, since the nutrition education intervention was conducted after school hours, full participation of these children was rather a challenge.

## Conclusions

The present study shows that NEI has no significant positive effects on overall nutrition knowledge or attitude of secondary schoolchildren and older adolescents. Since our findings showed no significant increase or improvement of nutrition knowledge and attitude scores of these secondary school children in our study, an in depth and different NEI which specifically tailored to the older or secondary schoolchildren need to be executed. To ensure the implementation and maintenance of the measures taken to combat obesity, including inculcation of positive attitude among older adolescents, continuous multi-pronged strategies and involvement from various sectors are required. A need for NEI inclusion in schools has been acknowledged.

## Data Availability

All relevant data are within the paper.

## Abbreviations

AMD: Adjusted mean difference
ICC: Intraclass/cluster correlation Coefficient
NEI: Nutrition education intervention
MDG: Malaysian Dietary Guidelines
NHMS: National Health and Morbidity Survey
IPH: Institute for Public Health
MyBFF@school: My Body is Fit and Fabulous @ school
